# Dynamic penetration behaviors of single/multi-layer graphene using nanoprojectile under hypervelocity impact

**DOI:** 10.1038/s41598-022-11497-x

**Published:** 2022-05-06

**Authors:** Weifu Sun, Tao Zhang, Jun Jiang, Pengwan Chen

**Affiliations:** 1 State Key Laboratory of Explosion Science and Technology, Beijing Institute of Technology, Beijing, 100081 China; 2 Beijing Institute of Technology Chongqing Innovation Center, Chongqing, 401120 China; 3 Explosion Protection and Emergency Disposal Technology Engineering Research Center of the Ministry of Education, Beijing, 10081 China

**Keywords:** Mechanical engineering, Graphene

## Abstract

Single/multilayer graphene holds great promise in withstanding impact/penetration as ideal protective material. In this work, dynamic penetration behaviors of graphene has been explored using molecular dynamics simulations. The crashworthiness performance of graphene is contingent upon the number of layers and impact velocity. The variables including residual velocity and kinetic energy loss under different layers or different impact velocities have been monitored during the hypervelocity impact. Results show that there exists deviation from the continuum Recht–Ipson and Rosenberg–Dekel models, but these models tend to hold to reasonably predict the ballistic limit velocity of graphene with increasing layers. Besides, fractal theory has been introduced here and proven valid to quantitatively describe the fracture morphology. Furthermore, Forrestal–Warren rigid body model II still can well estimate the depth of penetration of multilayer graphene under a certain range of velocity impact. Finally, one modified model has been proposed to correlate the specific penetration energy with the number of layer and impact velocity.

## Introduction

Because of the excellent properties, such as exceptionally high intrinsic strength and stiffness arising from the two-dimensional (2D) hexagonal lattice of covalently bonded carbon atoms^1^, electrical conductivity^2^, thermal conductivity^3^, since its discovery in 2004^4^, graphene with atomic monolayer thickness continues intriguing researchers^5,6^ and has broad engineering applications^7–14^. Especially, its inherent strength exceeding 100 GPa, Young’s modulus as high as 1 TPa^15,16^ and low density of 2.2 g/cm^3^ make multi-layer graphene (MLG) an extraordinary armor material exhibiting excellent impact energy delocalization under a supersonic penetration event and hold great promise in the field of safety protection for lightweight body armor design^17^ and spacecraft protection^18^. So far, most of experimental tests are restricted to the quasi-static mechanical properties of graphene except the recent supersonic projectile penetration carried out by Lee et al.^19^ in 2014, in which the high-strain-rate behavior of multilayer graphene by using miniaturized ballistic tests has been investigated. It has been discovered that the specific penetration energy for multilayer graphene is about 10 times more than literature values for macroscopic steel sheets^19^. Such extraordinary findings have aroused great interests in the application of graphene into the field of impact protection.

Confronted with experimental challenges, it is experimentally harsh to directly perform hypervelocity impact. As an alternative approach to physical experiments, molecular dynamics (MD) simulation^20^ has its unique advantages, which can be used to simulate hundreds of thousands or even millions of atoms by virtue of universally applicable force field relations. Therefore, MD simulation have provided much convenience and feasibility to understand the dynamic behaviors of 2D materials^21–26^. As a major two-dimensional material, there are lots of MD studies on graphene’s properties, especially its impact resistance. In terms of axial wave and cone wave propagation. Briefly, the failure mechanism of single-layer graphene under high-speed impact of diamond projectiles was explored by Xia et al.^24^ and found that the circular shape graphene possesses the best impact resistance for similar sample size. Furthermore, Bizao et al.^27^ simulated the process of 1–3 layers of graphene being subjected to the impact by 3.5–7.5 km/s of fullerene, evidencing the angle law of graphene crack observed from Lee’s experiment. It has been concluded that the high axial-wave and cone-wave velocity favor the momentum transfer and improvement of ballistic protection performance of graphene^23^. Meng et al.^22^ further explored the regularity of cone wave propagation relating to the boundary conditions and propose one approach to estimate the critical graphene membrane size, which offers theoretical guidance for the size engineering of graphene as a protective material. In addition, Bizao et al.^21^ put forward the law of scale effect to explain the discrepancy by one order of magnitude between experiments and MD simulations and correlate the specific penetration energy with the number of layers. In addition to graphene, other 2D material such as BC_3_and C_3_N whose structures is similar to graphene’s have also been explored by researchers using MD simulation^28–30^. Sadeghzadeh et al. explored the ballistic properties of C_3_N and BC_3_ nanosheets against hypersonic bullets with numbers greater than 6. The critical perforation conditions, and thus, the intrinsic impact strength of these 2D materials were determined by simulating ballistic curves of C_3_N and BC_3_ monolayers^30^. The mechanical properties of defective hybrid C_3_N–BC and defect-free hybrid C_3_N–BC_3_ also have been investigated. Results show that the effect of these flaws on Young’s modulus is less than their effect on failure stress and strain^29^. Further work has been performed to explore the mechanical and ballistic properties of Aluminum nanocomposites reinforced with monolayer polyaniline (C_3_N) and it is found that the nanocomposite can absorb 35% energy of nanoparticle when C_3_N was placed on the top of the aluminum surface^28^.

As we see, much progress has been made on the impact resistance of graphene and other 2D materials, nonetheless unresolved issues but of scientific importance remain unclear. The ballistic limit velocity is one of the important indicators signifying the material’s capability of impact resistance. Sadeghzadeh and Meng both found the result that the ballistic limit velocity increase with the increased number of graphene^22,31^, however, the inherent correlation between ballistic limit velocity and the number of layers of graphene are largely unknown. Is there a suitable way to predict the ballistic limit velocity of graphene at different layers? Are the continuum models related to penetration mechanics such as Recht–Ipson model^32^ still applicable at the ultra-small nanoscale? These need to be explored further. The description of the morphology of damaged graphene is usually qualitative and herein we make an effort to quantitatively to explain the morphology of the membrane after failure. What’s more, the residual velocity, kinetic energy consumption, impact displacement, total impact contact time and penetration depth in our work will be also discussed.

## Results and discussions

### Residual velocity and kinetic energy loss

**Figure 1:**
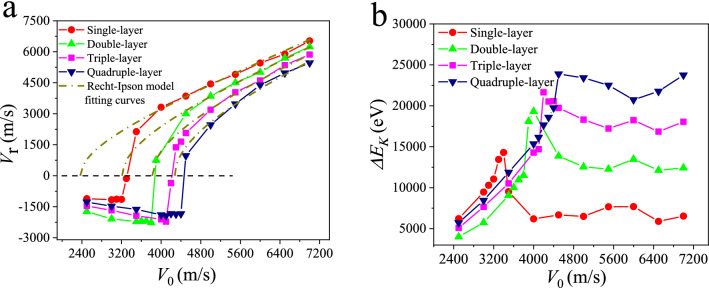
Residual velocity *V*_r_ (**a**) and kinetic energy loss Δ*E*_k_ (**b**) as a function of the initial impact velocity *V*_i_ of the projectile collided with single/multi-layer graphene, ranging from 2500 to 7000 m/s. The dashed dot line represents theoretical prediction from the continuum Recht–Ipson model. The minus symbol (−) of the residual velocity denotes the restitution process whereas the plus symbol (+) represents the penetration process.

In this part, the single or multi-layer graphene is subjected to the impact of the diamond nanosphere of 6.0 nm in diameter, acting as projectile, with an initial velocity *V*_0_ ranging from 2500 to 7000 m/s. The residual velocity *V*_r_, the kinetic energy loss Δ*E*_*K*_, i.e., $\Delta E_{K} = \frac{1}{2}m\left( {V_{0}^{2} - V_{r}^{2} } \right)$ as a function of initial impact velocity is illustrated in Fig. 1. Contingent upon the magnitude of the initial impact velocity *V*_0_, as discussed from Fig. S1 in Supplementary Information, different phenomena including ricochet without damage, ricochet with damage or penetration perforation occur. Correspondingly, with the increase of initial impact velocity, the residual velocity can be classified into three regions A, B and C as shown in Fig. S1 (see Supplementary Information), respectively. In region A, the residual velocity is negative, implying that the projectile does not perforate the single or multi-layer graphene and rebound back without damage to the graphene membrane. In contrast, in-between regions A and C, i.e., the transition region B, the projectile almost first gets stuck or embedded within the single layer graphene but finally rebounds back with the failure of the graphene membrane, and this critical initial impact velocity is defined as ballistic limit velocity *V*_bl_. The kinetic energy loss Δ*E*_k_ first increases with increasing the initial impact velocity to a peak value at around the ballistic limit velocity, followed by a drop and subsequent hike again as shown in Fig. 1b. Take the case of projectile of 4000 m/s impacting single-layer graphene for instance, the total energy consists of kinetic energy and potential energy. Part of the kinetic energy loss of projectile converts into the potential energy of graphene; another part of the kinetic energy loss of projectile convers into the kinetic energy of graphene, which can be reflected from the variation of the monitored temperature of graphene as shown in Figs. S2 and S3 in the Supplementary Information.

**Figure 2:**
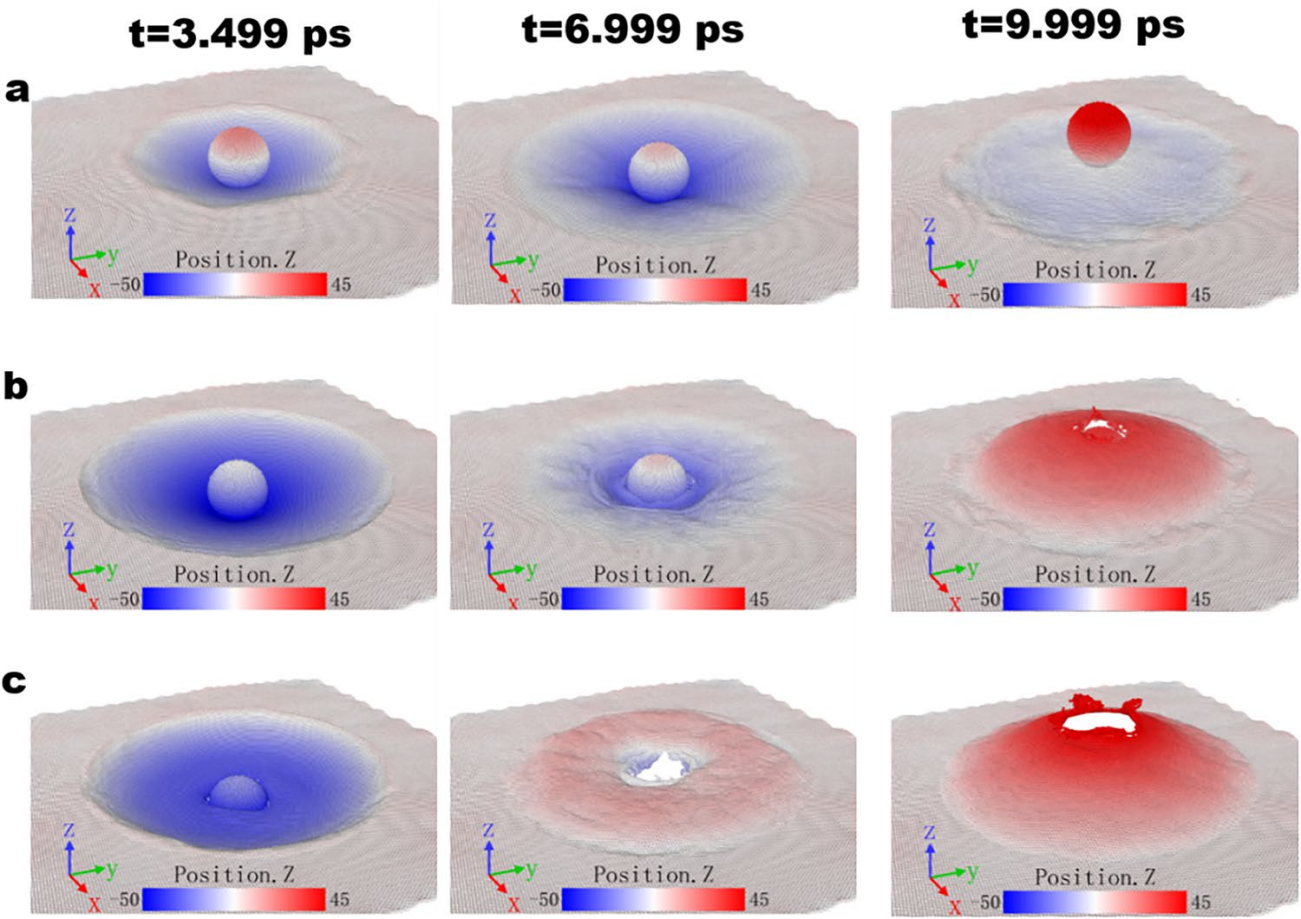
Simulation snapshots of diamond nano-projectile of 3.0 nm in radius colliding with single layer graphene at different time instants of 3.499, 6.999 and 9.999 ps with different initial impact velocities of 2000 ((**a**), ricochet without damage), 2750 ((**b**), ricochet with damage) and 3500 m/s ((**c**), perforation), respectively. Positions are identified and visualized by Ovito Version 2.9.0 (https://www.ovito.org/).

The simulation snapshots of projectile collided with the single layer graphene membrane at different initial impact velocities of 2000, 2750 and 3500 m/s are shown in Fig. 2. The snapshots in Fig. 2a extracted from the case of 2000 m/s corresponds to region A where restitution of the projectile occurs and there is no penetration while those in Fig. 2c extracted from the case of 2750 m/s corresponds to region C where the penetration of the projectile into the graphene happens. In contrast, the snapshots in Fig. 2b extracted from the case of 3500 m/s corresponds to region B where the restitution happens with the failure of the graphene membrane.

As the initial impact velocity rises from 2 to 7 km/s, the corresponding results of both *V*_r_ and Δ*E*_k_ present complicated and non-linear trends. The whole region can be roughly divided into three parts: Region A, B and C. To begin with Region A, the bounce-back, i.e., ricochet region, in which the initial velocity is comparably small, the projectile fails to perforate graphene and is bounced back without exerting damage to the graphene membrane. Under this circumstance, the membrane maintains a relatively good elastic deformation as shown in Fig. 2a. Furthermore, the magnitude of both the rebound velocity and kinetic energy consumption of the projectile increases with the increase of the initial impact velocity. Secondly, when it comes to Region B, the transition region, with the increase of impact velocity, the stress wave was bounced back upon reaching the fixed boundary, the convergence of which would generate an increase in membrane velocity that eventually exceeds the upward velocity of the bounced projectile. In consequence, the mutual extrusion effect between them results in a failure phenomenon in the center area of the membrane, as it is shown in Fig. 2b, i.e., ricochet with damage to the graphene membrane. Moreover, when the impact velocity is further increased, the residual velocity suddenly changes from negative to positive. The velocity range in which this transition occurs is relatively small. The projectile undergoes the rebounce back to the penetration of the membrane. During this process, the simulation result shows that the kinetic energy consumption has increased significantly. Finally, region C, or perforation region as shown in Fig. 2c, indicates that the projectile completely perforates the membrane. It can be seen that the residual velocity grows progressively with the increase of the initial velocity whereas the kinetic energy experiences a plateau period and afterwards gradually rises. The stress wave, as will be discussed in Fig. 4, was reflected from the boundary and converged in the central area. This will result in the higher velocity of the membrane than that of the upward-moving projectile and the mutual extrusion effect finally contributes to the failure of membrane. The corresponding interaction forces between projectile and target have been discussed in Fig. S4.

### The ballistic limit velocity *V*_bl_

Ballistic limit velocity is one of the important parameters in describing the anti-penetration capability of structures or materials. According to Recht–Ipson model^32^, the initial impact velocity *V*_0_, residual velocity and ballistic limit follow are correlated as follows,

1 $$V_{r} = \left( {\frac{{M_{P} }}{{M_{P} + m_{p} }}} \right)\;(V_{0}^{2} - V_{{{\text{bl}}}}^{2} )^{1/2} ,$$

where M_p_, m_p_ are the mass of projectile and the mass of plug ejected by projectile, respectively. When satisfying M_p_ ≫ m_p_, the above equation can be reduced to

2 $$V_{r} = (V_{0}^{2} - V_{{{\text{bl}}}}^{2} )^{1/2} .$$

In order to test the validity of the continuum model, a series of MD simulation have been performed by varying the initial impact velocity. Especially, a velocity increment of 10 m/s in the transitional region B is employed to accurately estimate the ballistic limit velocity. The theoretical predictions of ballistic limit velocity from the Recht–Ipson model for single layer to four layers are obtained by fitting the residual velocity to the Eq. (2), highlighted by the dashed dot lines as displayed in Fig. 1a.

As observed from Fig. 1a, the MD simulated residual velocities follows well with the Rech-Ipson continuum model. This indicates that the model is not only suitable for thin target on a macro scale, but also applicable for nanoscale penetration. Besides, the values of *V*_bl_ for single, double, triple and quadruple layer graphene are separately measured using the Recht–Ipson model and MD simulations and listed in Table 1. With the increase of the number of layers, the error percentage remains relatively low except the single and double layer graphene. Thus in a sense, the Recht–Ipson model provides a reliable approach to approximately predict the actual value of *V*_bl_ for nano-impact thin target, especially for larger thickness target.

**Table 1:** The number and proportion of lost atoms of single or multilayer graphene subjected to an impact velocity of 7000 m/s.

Membrane layer	Number of lost atoms	Number of original atoms in impact area	Loss percentage
Single-layer	215	1112	19.3
Double-layer	611	2233	27.3
Triple-layer	1087	3341	32.6
Quadruple-layer	1683	4477	37.5

Although the governing equation from Recht–Ipson model is simple and easy to use, nonetheless it is not associated with materials properties of the projectile and the target and lacks significant physical meaning. In order to further explore the ballistic limit velocity, we refer to the Rosenberg–Dekel model^33^, which employs the conception of effective resisting stress that simplifies complex penetration process, taking the form of

3 $$V_{{{\text{bl}}}} = \left( {\frac{{\sigma_{r} \cdot 2H}}{{\rho_{P} \cdot l_{eff} }}} \right)^{{1/2}} ,$$

where ρ_p_, H, σ_r_ are the density of projectile, the target thickness and effective resisting stress, respectively. *l*_eff_ is effective length of projectile obtained based on the principle of equivalent to a cylinder with equal cross-sectional area and mass.

According to the empirical formula provided by Rosenberg and Dekel^34^ MD, the ratio of σ_r_ and *Y*_t_ is related to the thickness of the target plate (H) and the diameter of the projectile (here D = 6 nm), *Y*_t_ is the strength of material. For the thin target (H/D < 1/3), there is:

4 $$\sigma_{r} /Y_{t} = 2/3 + 4\left( {H/D} \right).$$

**Figure 3:**
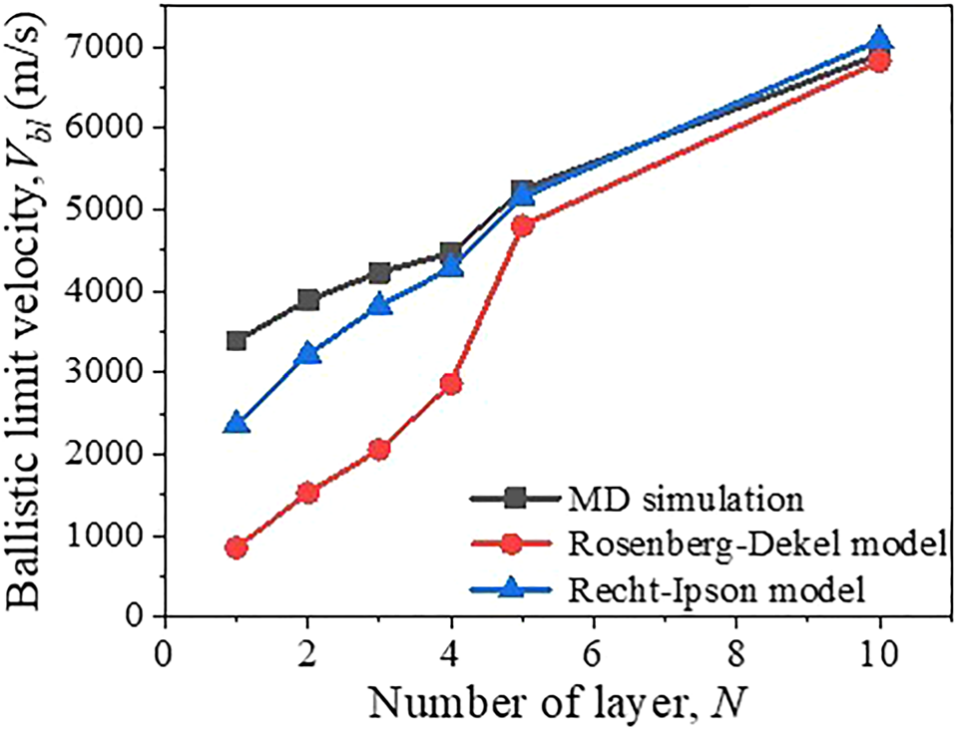
The ballistic limit velocity as a function of the number of layer of graphene obtained from MD simulations and theoretical predictions.

Equation (4) is not applicable for graphene to estimate the resisting stress, and so we turn to the direct estimation from MD simulations. The peak resisting stress is the key indicator to judge whether the graphene can resist the damage during the impact. The peak resisting stress can be obtained by the maximum resisting force divided by the corresponding contact area^35^. Normally, the maximum resisting force can be readily obtained by calculating the interaction forces between the projectile and target from the MD simulations. As for the corresponding contact area, it can be achieved with the aid of Materials Studio software, the corresponding surface area corresponding to the maximum resisting force can be calculated following the previous approach^36^, then the maximum impact stress is obtained. As per Eq. (3), the predicted *V*_bl_ can be calculated and then a comparison can be made with the simulated ballistic limit velocity from direct MD simulations. For single-layer graphene, we evaluate the peak resisting stress *σ*_*r*_ calculated under the impact of 3400, 4000, 5000, 6000 m/s, which are 15.3, 20.11, 38.16 and 42.92 GPa, respectively. It can be seen that for given number of layers, the peak resisting stress increases with the increase of impact velocity, which is consistent with the trend of peak stress as a function of velocity proposed by Meng et al. and Wu et al.^35,37^ in the process of graphene impact. Therefore, MD simulation can be used to obtain the critical impact velocity value close to or slightly greater than the ballistic limit velocity. Then, the calculated resisting stress can be employed using Eq. (3) to get *V*_bl_ with different layers from the Rosenberg–Dekel model, and finally a comparison can be obtained with those estimated from MD simulations as shown in Fig. 3 and the Table S1 in the Supplementary Information. It can be seen that the difference is very large when the number of layers is small, but when the number of layers is increased to 5 layers, 10 layers, the difference is reduced. It is understood that with the increase of the number of layers, the adaptability of the Rosenberg–Dekel model gradually hold for rigid penetration.

### The propagation of stress wave

**Figure 4:**
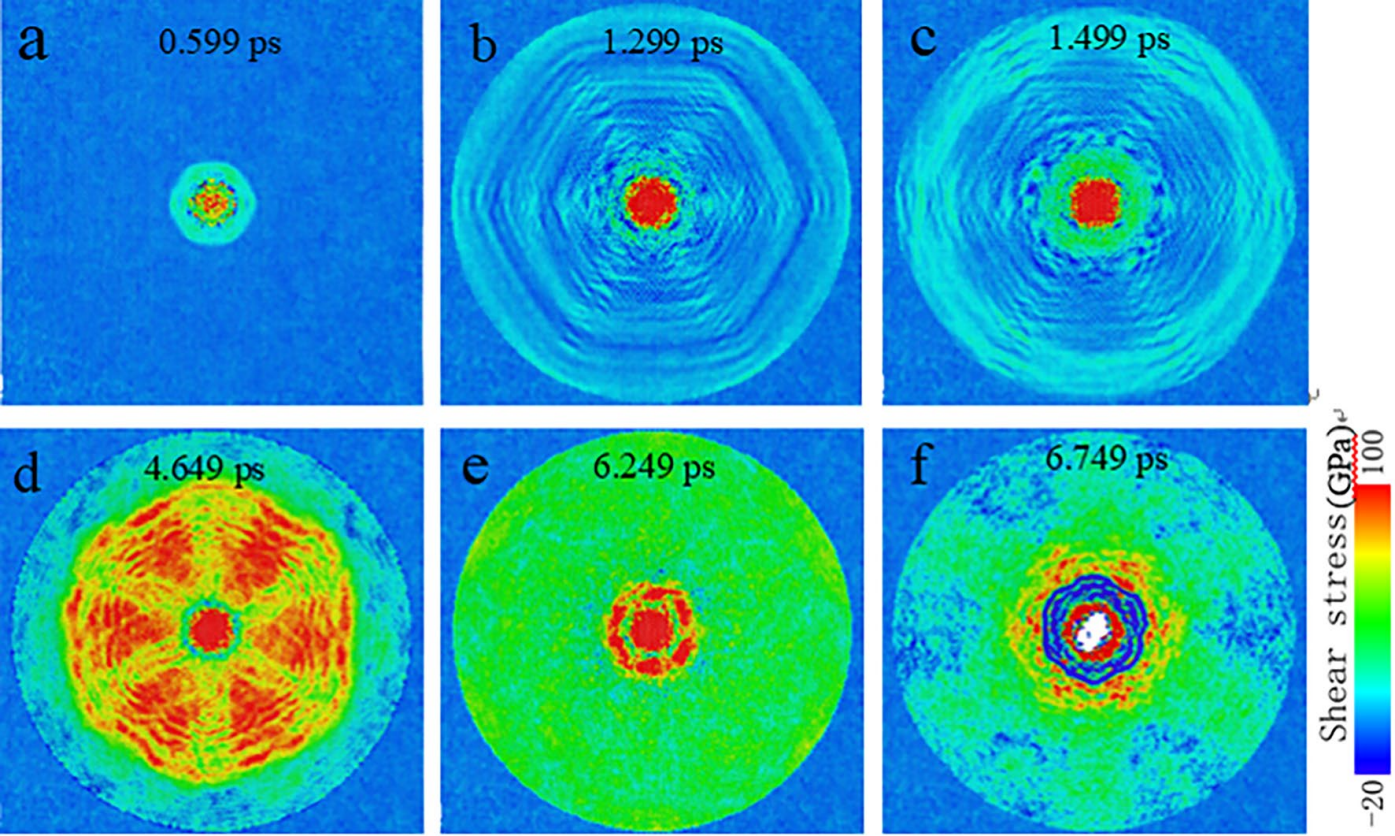
Propagation, reflection and convergence of stress waves in graphene membrane when subjected to the impact of diamond projectile at an initial impact velocity of *V*_0_ = 2750 m/s. Shear stress is identified and visualized by Ovito Version 2.9.0 (https://www.ovito.org/).

In order to demonstrate internal wave propagation and reveal the underlying mechanism of the failure of single layer graphene at 2750 m/s, shear stress distribution has been monitored at different simulation times. As observed from Fig. 4, at the very beginning at 0.599 ps, shear stress only focuses on a small area and propagates circumferentially; at 1.299 ps, stress wave transmits in the form of approximate hexagon rather than a circle. It is generally understood that the shock wave waveform should be circular since the boundary is set to be a fixed circular boundary, but we found that the waveform is hexagonal (Fig. 4). This phenomenon is similar to the results of Dong et al.’s research^38^, which is related to the graphene hexagonal structure and is caused by the different propagation speed of the shock wave along the armchair and zigzag directions. With the further increase of the time at 1.499 ps, the stress wave will be reflected until reaching the fixed boundary (border). The orange high stress area presents a hexagonal shape at 4.649 ps while the red central part represents higher stress caused by the compression between the membrane and the projectile. Interestingly, the border of the hexagon is the deformation bulge part of the membrane, which is just like a barrier converging stress waves to interact with each other within it. Subsequently, the hexagon area where shearing stress converges gradually shrinks to the size similar to the impact area until the strongest stress concentration appears at times of 6.249 and 6.749 ps. This can be vividly confirmed from the greatest resistance force exerting on the projectile by the graphene membrane as shown in Fig. S4b. The magnitude of the resistance force around 6.25 ps (> 1000 nN) is almost twice of those for the cases of “ricochet with/without damage”. Our results show that the graphene membrane might not be damaged at the initial impact, but can experience rupture even at smaller impact velocity than the ballistic limit velocity under the influence of strong stress concentration, facilitated by the fixed boundary. This also have been discovered by previous work conducted by Meng et al.^22^.

### Morphology, failure mechanism and mass loss of the damaged graphene

It is of scientific importance to investigate the morphology of hole left in the graphene membrane after penetration and also the corresponding mass loss. In view of the irregularity of the holes after destruction, the shape factor in the fractal theory (see Fig. S5 in the supplementary information) has been employed here to explore the latent regularities. The top views of the final hole at different impact velocities are shown in Fig. S6. The hole shape gradually transforms from triangle, quadrangle, pentagon and then to hexagon and the number of petals also gradually increases, all of these are consistent with the results reported^27^.

**Figure 5:**
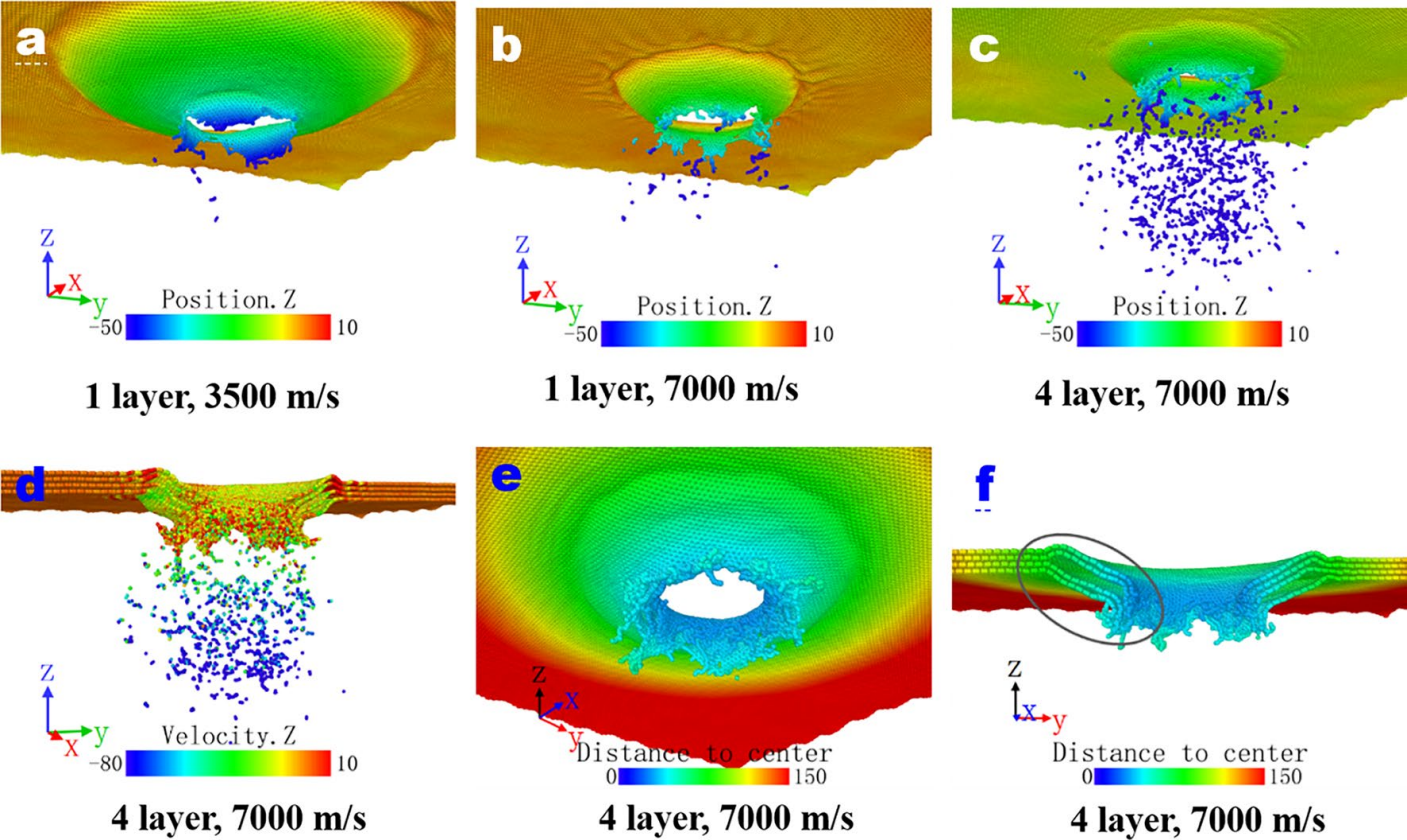
The final morphologies of single-layer graphene subjected to impact velocities of 3500 m/s (**a**) and 7000 m/s (**b**) respectively; the position (**c**) and velocity (**d**) along the Z-direction position, bottom view (**e**) and cross-section view (**f**) of the quadruple-layer graphene membrane at 7000 m/s. Distances are identified and visualized by Ovito Version 2.9.0 (https://www.ovito.org/).

Failure modes of the single or multi-layer graphene membrane have been analyzed through the final configuration or morphology by considering the factors including the number of layer and the magnitude of impact velocities. As observed from Fig. 5a,b, the sing layer graphene result in 3 and 6 petals, respectively, after being impacted by the projectile at 3500 and 7000 m/s while the size of the former petal and impact area are larger. Herein the main form of graphene membrane failure is the radial extending, and petal-shaped failure occurs so long as the tensile stress reaches the failure stress. Similar to the “debris particle cloud”, a lot of lost atoms can be observed. The number of lost atoms also increases with increasing the impact velocity. Likewise, as observed from Fig. 5b,c, the number of lost atoms increases with increasing the number of layer for given identical impact velocity of 7000 m/s. Besides, velocity contour (Fig. 5d) show that atomic particles at the bottom are rushed out at a high velocity close to the projectile speed whereas the speeds of the atomic particles on both sides are slightly lower, which resembles the phenomenon of debris particle cloud under hypervelocity impact. As confirmed by the bottom and cross-sectional view (Fig. 5e,f) of the damage structure of graphene membrane, similar to the form of plugging damage, when both the number of layers and the impact velocity are large, the deformation mainly occurs just in small impact area and the incomplete radial extension after the penetration has ended, which signifies a shift of deformation from global to local. The impact area is damaged by the action of strong radial shearing force under hypervelocity impact. Consequently, the failure mode of graphene under high speed impact manifests a more complicated situation, which involves “debris particle cloud” and plugging.

An interesting phenomenon of the increasing number of lost atoms with increasing the number of layer has been displayed. It is of scientific importance to do the statistics of the number and proportion of lost atoms from the final configuration for the cases of single, double, triple and quadruple-layer graphene at impact velocity of 7000 m/s (Table 1). It is found that, in effect, even if all the impacts are carried out at the same speed of 7000 m/s, the percentage of lost atoms for different layers are different and exhibits an increasing trend with respect to the number of layer. This is closely related to the radially propagating stress, which firstly arrives at the edge of the graphene, followed by a reflected reverse unloading wave. This wave will interact with the later compression stress wave to produce a tensile effect at the junction (Fig. 5f). A large gap between layers, i.e., spalling, can be observed (Fig. 5f) because of the wave interaction, which also push the lost atoms forward with a high speed. This can also be explained in terms of the lost atoms. The lost atoms generated from the top or former layer will act as newly emerging nano-projectile to impact with the down/latter layer to create more lost atoms.

### Depth of penetration of multilayer grapheme

Depth of penetration (DOP) is an important indicator of impact resistance, therefore it becomes necessary to accurately estimate this parameter using MD simulations and test the validity of the continuum penetration depth models of rigid projectile at the atomic/nanoscale. For rigid projectile, Forrestal–Warren (F–W) model^39^ and Rosenberg–Dekel (R–D) model^40^ are widely used in describing the penetration depth.

#### F–W model I

5 $$P/L = \frac{1}{{3N^{*} }}\left( {\frac{{\rho_{p} }}{{\rho_{{\text{t}}} }}} \right)\ln \left( {1 + \frac{{3N^{*} \rho_{t} }}{{2\sigma_{yt} }}V_{0}^{2} } \right),\;N^{*} = \frac{8\psi - 1}{{24\psi^{2} }},$$

where *P, L*, *ρ*_*P*_ and *ρ*_*t*_ are the penetration depth, effective length of projectile, densities of projectile and target, respectively; *σ*_yt_ is the quasi-static yield strength of target, *ψ* = 0.5 for the hemispherical nosed projectile and thus the projectile nose coefficient N^*^ = 0.5. *L*, *ρ*_*P*_, *ρ*_*t*_ and *σ*_yt_ are 4 nm, 3.5 g/cm^3^, 2.2 g/cm^3^, 130 GPa, respectively.

#### F–W model II

F–W Model II is the first-order Taylor expansion of F–W Model I, which is equivalent to the upper limit of the F–W model. Parameter $3N^{*} \rho_{{\text{t}}} V_{0}^{2} /2\sigma_{yt}$ in Eq. (5) is small as compared with unity for a practical range of striking velocity, thus performing a first-order Taylor expansion at 0, so Eq. (5) can be rewritten as:

6 $$P/L = \frac{{\rho_{{\text{p}}} }}{{2\sigma_{{{\text{yt}}}} }}V_{0}^{2} ,$$

where *ρ*_*P*_ and *σ*_yt_ have the same meanings and values as them in F–W model I.

#### R–D model

7 $$P/L = \frac{{\rho_{P} V_{0}^{2} }}{{2\sigma_{ft} [1.1 \times \ln ({{E_{t} } \mathord{\left/ {\vphantom {{E_{t} } {\sigma_{ft} }}} \right. \kern-\nulldelimiterspace} {\sigma_{ft} }}) - \Phi ]}},$$

where Φ = 0.2 for the hemispherical nosed projectile, *E*_*t*_, *σ*_*ft*_ are the elastic modulus and flow stress of the target, which are 1 TPa and 140 GPa, respectively, for the graphene.

**Figure 6:**
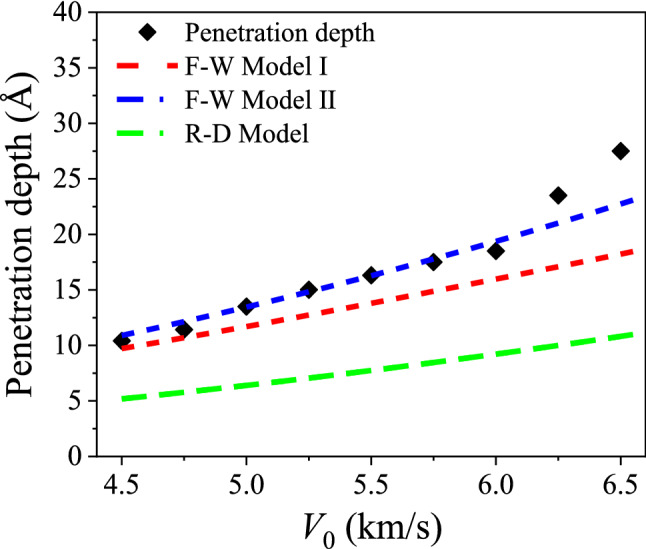
Comparison of penetration depth of ten-layer graphene obtained from MD simulations and continuum model predictions.

Since these theoretical models require that the shape of the projectile should be hemispherical nosed projectile. As per the principle of equal cross-sectional area and mass, the 6 nm rigid spherical projectile is equivalent to one hemispherical nosed projectile with a length of 2.0 nm and radius of 3.0 nm, as shown in Fig. S7. In order to test the validity of the continuum models, appropriate range of impact velocity ranging from 5250 to 8000 m/s have been chosen for 10 layer graphene. Because if the impact velocity is too small, only elastic deformation will occur without penetration; if the impact velocity is too high, the membrane will be directly penetrated through. Only when the impact velocity falls within certain range, plastic deformation will happen without penetration. The depth of plastic deformation of the projectile without penetration is defined as the penetration depth. A comparison of depth of penetration obtained from MD simulated results and theoretical predictions including F–W model and R–D model has been made (Fig. 6). The MD simulated DOP is much higher than those from R–D model, but reasonably agree with F–W model I, especially at impact velocity smaller than 7000 m/s. As the initial impact velocity further increases to more than 7000 m/s, the MD simulated ones will deviate from the F–W model I. This can be explained as follows: Because of the inherently limited number of layers, the bottom layer is prone to be easily deformed at high impact velocity, resulting in a sharp increase in the penetration depth after 7250 m/s. This becomes evident as the impact velocity is close to the ballistic limit velocity. In general, the F-–W model still can quantitatively estimate the penetration depth of a hemispherical rigid projectile impacting multi-layer graphene at the ultra-small nanoscale. Note that the Forrestal–Warren perforation model has been experimentally proved valid in predicting the DOP for a range of “rigid” steels including high hardness steel armour and Ti–6Al–4V^41^. Besides, the total impact displacement, which is the distance of the projectile travelling from the initial contact point in the approach process to the final contact point at the instant of initiated restitution where the kinetic energy becomes zero or perforation along the Z-axis. Correspondingly, the total time of impact is the time interval from the instant of the initial contact to that of the final contact between the projectile and graphene. These two parameters have also been monitored as shown in Fig. S8.

### Specific penetration energy

**Figure 7:**
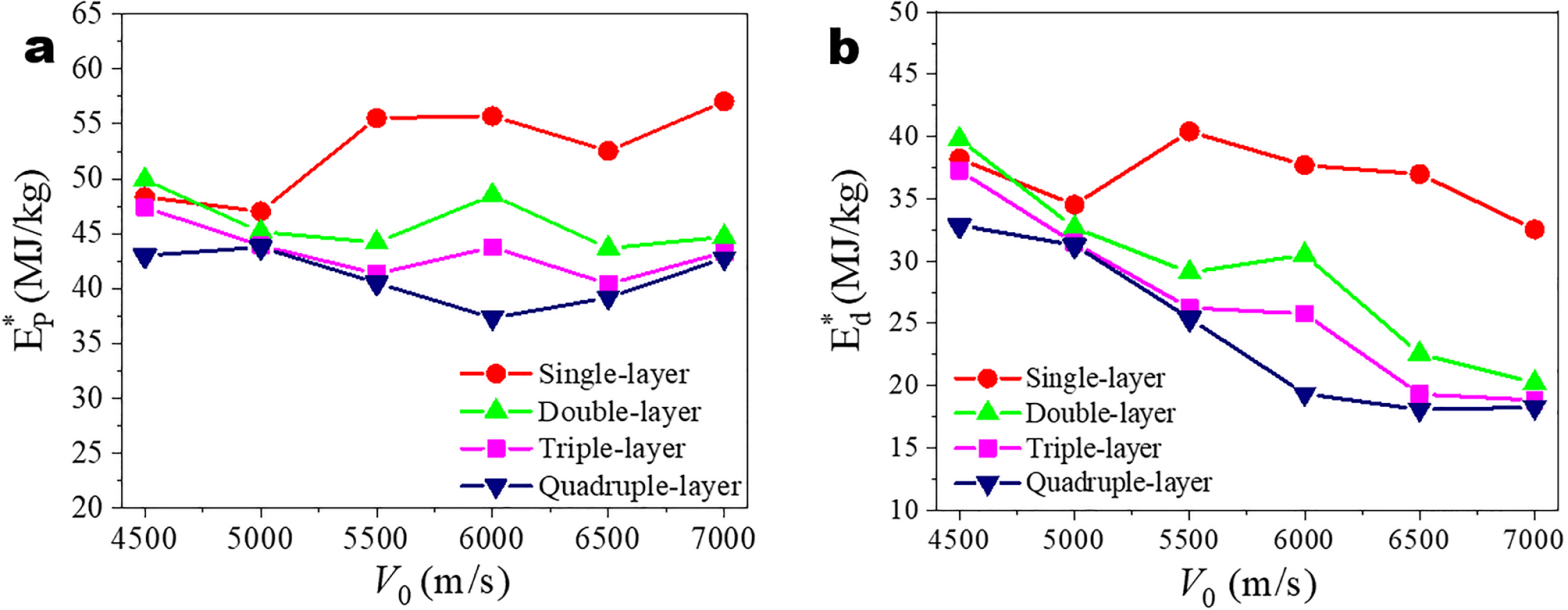
$E_{P}^{*}$ (**a**) and $E_{d}^{*}$ (**b**) of single or multi-layer graphene as a function of initial impact velocity.

Lee et al.^19^ proposed the following relationship as per their experimental results,

8 $$E_{P}^{*} = \frac{1}{2}v_{0}^{2} + E_{d}^{*} ,$$

where $E_{P}^{*}$, $E_{d}^{*}$ are the specific energy dissipation and the specific delocalized penetration energy. $E_{d}^{*}$ is an indicator to evaluate the impact energy delocalization ability of the partial material beyond direct compact region of the material as more sample mass beyond direct compact region contributes to the energy dissipation. $E_{P}^{*}$ and $E_{d}^{*}$ for different layer graphene at different impact velocities are calculated and shown in Fig. 7. The magnitude of $E_{P}^{*}$ is on the order of several tens ranging from 20 to 50 MJ/kg for a couple of layers only, which is much higher than those reported experimental results of 1.1 and 1.3 MJ/kg at 600 and 900 m/s, respectively, for the 300 layers of graphene with a thickness of 100 nm^19^. This has been discovered by Rafael’s research^21^ and the value of $E_{P}^{*}$ is affected by the scale of the model (i.e., the size of graphene). Rafael explained the rationality of the difference from the viewpoint of the scale effect relationship. Note that our simulated value of $E_{P}^{*}$ is similar to those obtained by Bizao^21^ and basically consistent with the results of Haque^27^. For instance, at impact velocity of 4500 and 5000 m/s, Haque accordingly obtained the $E_{P}^{*}$ of 38.44 MJ/kg and 42.65 MJ/kg, respectively, which are on the same order of magnitude as obtained here. This actually provides support and confidence in the reliability of our simulations. Besides, as observed from Fig. 7b, $E_{d}^{*}$ is dependent not only upon number of layer, but also upon the impact velocity: with the increase of impact velocity (that is greater than ballistic limit velocity), each layer’s $E_{d}^{*}$ exhibits a decreasing trend; for given impact velocity, $E_{d}^{*}$ decreases with increasing the number of layer. This indicates that the proportion of energy absorption outside the impact area decreases and explains the increasing regularity of the impact region and the rise in the number of lost atoms. Xia et al. performed simulation of a spherical projectile impacting a single-layer graphene film with different shapes, the specific penetration energy obtained increases with the increase of impact speed, which is consistent with our results. Besides, the value of the specific penetration energy of the circular graphene film is on the same order of magnitude as the result of our simulation^42,43^.

Although scale law has been put forward in terms of number of layer in Rafael’s research, nonetheless the influencing factor of impact velocity has not been properly considered. In order to further accurately predict $E_{d}^{*}$ and correlate $E_{d}^{*}$ with the governing factors, the following exponential function has been constructed to fit the available simulation and experimental results:

9 $$E_{d}^{*} \left( {N,\frac{{V_{0} }}{{V_{{{\text{bl}}}} }}} \right) = E_{d}^{*} \left( {\infty ,\frac{{V_{0} }}{{V_{bl} }}} \right)\sqrt {1 + \frac{{N_{C} }}{{N + N_{C}^{^{\prime}} }}} \, \left[ {1 + Ae^{{B\left( {\frac{{V_{0} }}{{V_{bl} }}} \right)}} } \right],$$

where *N*, $V_{0} /V_{bl}$ are the number of layer and the ratio of initial impact velocity *V*_0_ to the ballistic limit velocity *V*_bl_; *N*_*C*_, *N*^*’*^_*C*_, *A*, *B* are the coefficients as obtained from the best fitting.

**Figure 8:**
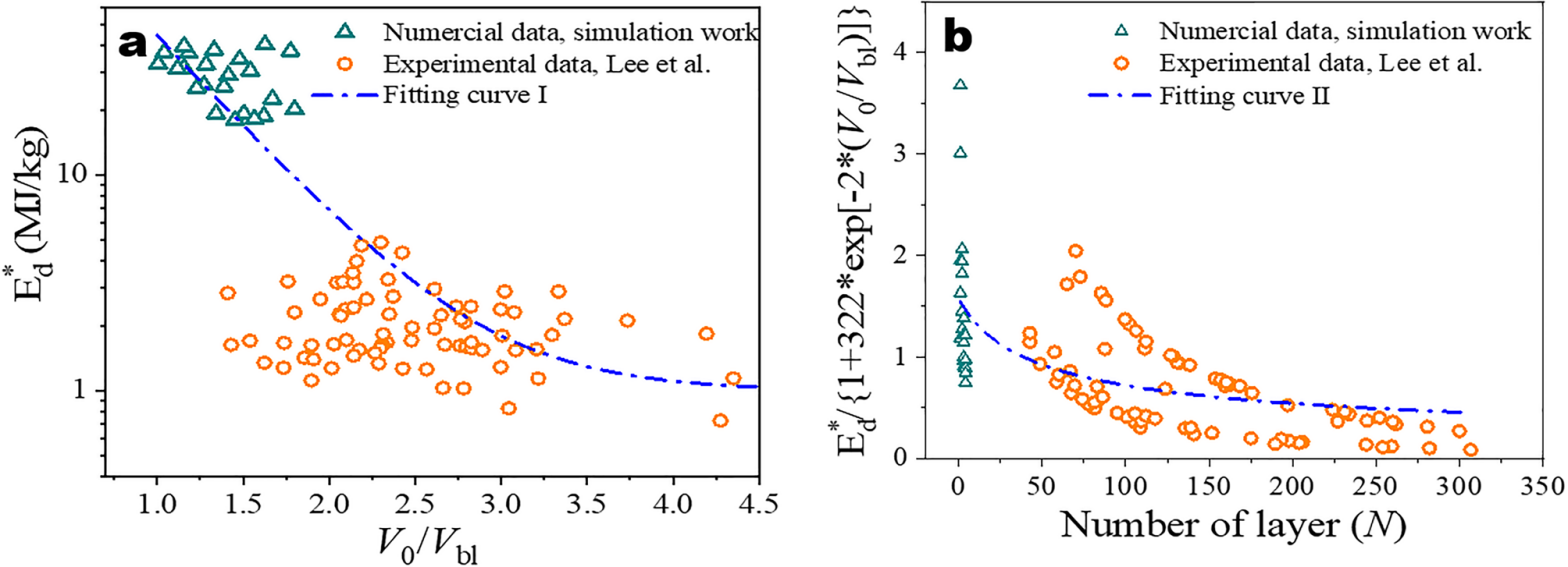
Specific penetration energy $E_{d}^{*}$ as a function of normalized initial impact velocity by ballistic limit velocity, *V*_0_/*V*_bl_ and normalized specific penetration energy as a function of the number of graphene layer *N*.

In order to obtain one reliable and representative relationship, a number of experimental data from Lee et al.^19^ have been re-drawn and incorporated along with the simulated data for the fitting as displayed in Fig. 8. Since the ballistic limit velocity is contingent upon the number of layer, so the specific penetration energy versus the normalized initial impact velocity was first fitted to the proposed function of $A\exp \left[ {B({{V_{0} } \mathord{\left/ {\vphantom {{V_{0} } {V_{bl} }}} \right. \kern-\nulldelimiterspace} {V_{bl} }})} \right]$, the as-obtained A and B are 322 and − 2, respectively; then the normalized specific penetration energy was fitted to the $E_{d}^{*} (\infty ,\;V_{0} /V_{bl} )\sqrt {1 + N_{C} /\left( {N + N_{C}^{^{\prime}} } \right)} \,$. Finally, fitting results show *N*_*C*_*, N*^*’*^_*C*_*, A, B* are 6385, 25.89, 322, − 2, respectively. Then Eq. (10) can be rewritten as

10 $$E_{d}^{*} \left( {N,\frac{{V_{0} }}{{V_{bl} }}} \right) = 0.1\sqrt {1 + \frac{6385}{{N + 25.89}}} \, \left[ {1 + 322e^{{ - 2\left( {\frac{{V_{0} }}{{V_{bl} }}} \right)}} } \right].$$

Finally, note that the results we reported are directly obtained from MD simulation since the reported simulations give rise to basically the same results. There are other factors that can affect the simulation results, such as the surface roughness of the nanospheres. This factor was also explored in our previous work on the high-speed impact of two nanospheres^36^. We have further explored the influence of nanoprojectile’s surface roughness by rotating the diamond nanospheres along the Z axis by 45°, 90°, 135°, and 180°. The results as shown in the Figs. S9 and S10 in the Supplementary Information indicate that the surface roughness of nanospheres has a small effect on impact simulations, which is similar to the conclusions of previous work^36,44^.

## Conclusions

In this work, the typical indicators associated with dynamic penetration behaviors including ballistic limit velocity, depth of penetration and specific penetration energy of single/multi-layer graphene have been explored using fully atomistic molecular dynamics simulations and the validity of relevant continuum models such as Recht–Ipson model, Rosenberg–Dekel laminated model, Forrestal–Warren model and Rosenberg–Dekel model have been tested at the nanoscale. The results show that during the process of diamond nano-projectile impacted with single/multilayer graphene, the penetration phenomena including ricochet without damage, ricochet with damage or penetration perforation occur, which is dependent upon the magnitude of initial impact velocity. The simulation process, internal stress wave propagation and interaction forces between nano-projectile and graphene have been monitored and analyzed to explain such phenomenon transition. The graphene membrane even can be damaged at smaller impact velocity than the ballistic limit velocity under the influence of strong stress concentration and extrusion effect. The residual velocity first decreases and then increases with increasing the initial impact velocity whereas the kinetic energy loss largely follows the opposite trend. The critical initial impact velocity corresponding to the ricochet with damage to the graphene is defined as the corresponding ballistic limit velocity *V*_bl_. There exists deviation from the continuum Recht–Ipson and Rosenberg–Dekel models, but these models tend to hold to reasonably predict the ballistic limit velocity of graphene with increasing layers.

The morphology of hole left in the graphene membrane after perforation and also the corresponding mass loss have been monitored. The hole shape gradually transforms from triangle, quadrangle, pentagon and then to hexagon and the number of petals also gradually increases with increasing impact velocity as confirmed by the shape factor in the fractal theory, which has been adopted in quantitatively describing the irregularity of the holes after destruction. The failure modes of graphene under high speed impact are associated with “debris particle cloud” and plugging. Besides, the maximum impact displacement and total time of impact from the initial contact to the detachment have also been monitored from MD simulations. The depth of penetration of graphene under the impact of diamond nano-projectile has been measured using MD simulations. Rosenberg–Dekel model delivers a much lower estimate but Forrestal–Warren II model still holds to predict the depth of penetration at the nanoscale. Generally, the specific penetration energy of single/multilayer follows a monotonic decreasing function of both the number of layer of graphene and the initial impact velocity. One newly modified equation has been proposed to correlate the specific penetration energy with the initial impact velocity apart from the number of layer of graphene.

## Simulation methods and condition

In this paper, the MD simulation processes are performed using the Large-scale Atomic/Molecular Massively Parallel Simulator (LAMMPS)^45^ and the simulation results and snapshots are acquired with the assistance of visualization software OVITO (Version 2.9.0)^46^. The projectile is made of diamond nanosphere with a diameter of 6 nm, which is built by cutting from the diamond structured diamond with the lattice constant of 0.357 nm bulk using LAMMPS. The mass of the projectile containing 19,889 carbon atoms is 2.01 × 10^–22^ kg. Single layer, double-layer, triple-layer and quadruple-layer graphene are built using Visual Molecular Dynamics (VMD)^47^ with a dimension of 40 nm × 40 nm in x, y axes, respectively. The adaptive intermolecular reactive empirical bonder order (AIREBO) potential was adopted herein since it has been proved valid to describe the interaction between C–C and C–H atoms^48,49^ and reproduce the mechanical properties of carbon systems such as graphene^24,27^. But it is suitable for describing the system containing graphene and diamond. Therefore, for the interaction between graphene and diamond, we refer to previous studies and set the LJ parameter separately to describe the interaction between graphene and diamond^22,37^. Because, although both graphene and diamond are composed of carbon atoms, their atomic arrangement and structure are completely different. Besides, because of the high Young’s modulus of diamond and for the sake of comparison with the “rigid” assumption inherent in continuum model, the treatment of diamond projectiles as rigid bodies in the nano-scale impact process has also been adopted herein and elsewhere^22,24,27,37,50–53^. The effect of rigid assumption has been evaluated as shown by Figs. S11 and S12 in the in the Supplementary Information. Briefly, single or multi-layer graphene was fully relaxed using NVT ensemble running for at least 500 ps at 10 K to obtain a stable graphene structure with minimized energy. The formation of wrinkles around the graphene can be observed after energy minimization, which is consistent with the results reported elsewhere^54^. The projectile is regarded as a rigid body^22,24^ The interaction between graphene and projectile is described by the 12-6 Lennard Jones (LJ) potential and the values of ε_LJ_, σ_LJ_ are 0.035 eV and 3.46 Å^55^, respectively. Note that the boundary of graphene is fixed while the central circular area of 15.0 nm in radius is unconstrained. Since graphene is a two-dimensional and thin material, the entire graphene sheet will move at high speed when subjected to the collision with the projectile. In this case, it is difficult to further study the impact resistance of graphene. Thus, one feasible but carefully corroborated approach of fixed boundary has been adopted herein and the detailed validation process can refer to the Supplementary Information. The projectile is perpendicularly located about 10 nm apart from the center of the graphene membrane. An initial impact velocity ranging from 2000 to 7000 m/s was applied to projectile and the whole impact process is simulated using NVE ensemble with a time step of 0.5 fs^24,27,43^.

Note that penetration can be classified into rigid penetration, deformation penetration and eroding penetration^40^, and the corresponding continuum theories are also different. Our paper mainly is associated with the rigid penetration, with a focus on the impact of “rigid” projectile with the graphene. The effect of “non-rigid” projectile and influence of “free” or “fixed” boundary conditions have also been provided in the Supplementary Information. The setting of “free” or “fixed” boundary conditions will affect the shockwave reflecting, but has little influence on the main conclusion regarding the penetration behaviors of multilayer graphene in this work.

## Supplementary Information

Supplementary Information.

## Data Availability

The data that support the findings of this study are available from the corresponding author (W. F. Sun) upon reasonable request.
