# Supplementary material for: Dynamic penetration behaviors of single/multi-layer graphene using nanoprojectile under hypervelocity impact
Source: Sci Rep. 2022 May 6;12:7440. doi: 10.1038/s41598-022-11497-x (PMC9076916; doi:10.1038/s41598-022-11497-x)
Supplement: Supplementary file 1 — Supplementary Information. [file 41598_2022_11497_MOESM1_ESM.docx]

**Dynamic penetration behaviors of single/multi-layer graphene using nanoprojectile under hypervelocity impact**

**Supplementary information**

Weifu Sun^1,2,3,🖂^, Tao Zhang^1,3^, Jun Jiang^1,3^, Pengwan Chen^1,3^

^1^ State Key Laboratory of Explosion Science and Technology, Beijing Institute of Technology, Beijing 100081, China. ^2^ Beijing Institute of Technology Chongqing Innovation Center, Chongqing, 401120, China. ^3^ Explosion Protection and Emergency Disposal Technology Engineering Research Center of the Ministry of Education, Beijing 10081, China. ^🖂^Corresponding author E-mail: [weifu.sun@bit.edu.cn](mailto:weifu.sun@bit.edu.cn).Tel/Fax: +861068912696.

**Results and discussion**

1. The critical size of the graphene

The validity of Recht-Ipson model needs to satisfy the following conditions that the projectile is rigid or slightly deformed during penetration; the transverse surface of the membrane should be infinite whereas the thickness of the target plate could be finite. It has been proved in previous studies, especially, it is also applicable to thin targets with small ratio of target thickness to the diameter of projectile (H/D<1/2).

It is worth noting that the theoretical model requires that the transverse surface is an infinite area, which means that the stress wave caused by the impact propagates continuously outwards. Actually, in Lee’s experiment[^1^](#_ENREF_1), the size is finite. In our simulation, the graphene is also finite in size and the boundary is fixed, that is to say, the stress wave cannot propagate continuously outwards, and will experience the boundary reflection. In previous studies, it was reported that the reflected cone wave would reduce the ballistic limit velocity of the membrane. So there is a doubt: whether the ballistic limit velocity is reduced and whether the Recht-Ipson model can be applicable under our model size and boundary condition?

Originally, much effort has been made to apply periodic boundary conditions to the graphene sheet during the impact process. Since graphene is a two-dimensional and thin material, the entire graphene sheet will move at high speed when subjected to the collision with the projectile. In this case, it is difficult to further study the impact resistance of graphene. Thus, one feasible but carefully corroborated approach of fixed boundary has been adopted herein.

After the graphene boundary is fixed, the reflected wave generated at the fixed boundary may have an impact on the ballistic limit velocity, so the size of the graphene is a key factor. Meng et al.[^2^](#_ENREF_2) explored the regularity of cone wave propagation relating to the boundary conditions and propose one approach to estimate the critical graphene membrane size, which offers theoretical guidance for the size engineering of graphene as a protective material. A critical size relationship is given by Meng et al.[^2^](#_ENREF_2):

$$a_{c}=\frac{k_{2}}{k_{1}}r_{p}^{3/2}(\frac{\rho_{p}}{\rho_{m}})^{1/2}(\frac{1}{h_{m}})^{1/2}\text{，}$$

- (1)

where r_p_, ρ_p_ are the diameter and the density of projectile, r_m_, ρ_m_ are the thickness

and density of graphene, *k*_1_ and *k*_2_ are coefficients. This formula shows that when the size of the projectile is determined and when the model radius *r*_c_ is smaller than *a*_c_, the cone wave will significantly reduce the ballistic limit velocity, otherwise, there will be no obvious effect. Since *a*_c_ is inversely proportional to$h_{m}^{1/2}$, if the radius of single layer can satisfy r_c_>a_c_, then more layers can also satisfy. Bring the relevant parameters of the projectile and the single-layer membrane (r_p_=3nm, ρ_p_=3.5 g/cm^3^, ρ_m_=2.2 g/cm^3^, h_m_=0.335 nm, k_2_/k_1_=0.96~1.17) into Eq. (3) and obtain a_c_ lying between 10.87~13.20 nm, which is smaller than ***r*_c_ (*r*_c_=15 nm)**. In this work, the diameter of graphene is 30 nm, this shows that the size of the fixed boundary model in our simulation has no significant effect on the ballistic limit velocity *V*_bl_, then it can be regarded as "infinite".

We varied the size of graphene and determine the critical size by exploring on the value of V_bl_. For monolayer graphene, different impact zone sizes with a diameters of 20 nm, 25nm, 30nm and 35nm have been employed, respectively. The range of ballistic limit velocity was roughly simulated and calculated, as shown in **Table S1**.

**Table S1** The range of ballistic limit velocity of different impact zone sizes of graphene.

| Size (nm) | *V_0_* (m/s) | *V_r_* (m/s) | State of graphene | *V_bl_* (m/s) |
| --- | --- | --- | --- | --- |
| 20 | 2700 | -315 | ricochet with damage | 2740 $\pm$10 |
|  | 2800 | 115 | perforation |  |
| 25 | 3000 | -563 | ricochet with damage | 3060 $\pm$10 |
|  | 3100 | 403 | perforation |  |
| 30 | 3300 | -103 | ricochet with damage | 3390$\pm$10 |
|  | 3400 | 39.6 | perforation |  |
| 35 | 3400 | -1010 | ricochet with damage | 3420 $\pm$10 |
|  | 3500 | 1772 | perforation |  |

It can be seen that the size of graphene with a diameter of 20 nm and 25 nm has a great influence on the ballistic limit velocity. When it reaches 30 nm and 35 nm, it is found that the difference of ballistic limit velocity becomes basically small, within the acceptable error range, which is basically consistent with the results of Meng et al. This shows that there is a critical size, beyond a certain size, the influence of fixed boundary on ballistic limit velocity will be negligible. In addition, in many simulations of projectiles impacting graphene, fixed boundary conditions of graphene have been adopted, so that the dynamic penetration behaviors of graphene can be explored using MD simulations [^3-8^](#_ENREF_3).

1. **Residual velocity and kinetic energy lost for single layer graphene**

Generally speaking, when the projectile is impacted with the target, the projectile will experiences three different phenomena, which are called “embedment”, “perforation” and “ricochet”. But at the ultrasmall nanoscale, when a nanoprojectile is impacted with the layered membrane, the following phenomena including “ricochet without damage”, “ricochet with damage” and “perforation” have been observed from our molecular dynamics (MD) simulations.

When the single-layer graphene is impacted by the nanoprojectile at different initial impact velocities (1 km/s~7 km/s), the relationship among residual velocity *V*_r_, kinetic energy consumption *ΔE*_k_ and initial impact velocity *V*_0_ is obtained as shown in **Fig. S1**. As the initial impact velocity rises from 1 km/s to 7 km/s, the corresponding results of both *V*_r_ and *ΔE*_k_ present complicated and non-liner trends. The whole region can be roughly divided into three parts: Region A, B and C. To begin with Region A (1km/s~2.25 km/s), the bounce-back, i.e., ricochet region, in which the initial velocity is comparably small, the projectile fails to perforate graphene and is bounced back without exerting damage to the graphene membrane. Under this circumstance, the membrane maintains a relatively good elastic deformation as shown in **Fig. 2(a)**. Furthermore, the magnitude of both the rebound velocity and kinetic energy consumption of the projectile increases with the increase of the initial impact velocity. Secondly, when it comes to Region B (2.25km/s~3.25 km/s), the transition region, with the increase of impact velocity, the stress wave was bounced back upon reaching the fixed boundary, the convergence of which would generate an increase in membrane velocity that eventually exceeds the upward velocity of the bounced projectile. In consequence, the mutual extrusion effect between them results in a failure phenomenon in the center area of the membrane, as it is shown in **Fig. 2(b),** i.e., ricochet with damage to the graphene membrane. Moreover, when the impact velocity is further increased, the residual velocity suddenly changes from negative to positive. The velocity range in which this transition occurs is relatively small. The projectile undergoes the rebounce back to the penetration of the membrane. During this process, the simulation result shows that the kinetic energy consumption has increased significantly. Finally, region C（>3.25 km/s）, or perforation region as shown in **Fig. 2(c)**, indicates that the projectile completely perforates the membrane. It can be seen that the residual velocity grows progressively with the increase of the initial velocity whereas the kinetic energy experiences a plateau period and afterwards gradually rises.

**Figure. S1** Residual velocity *V*_r_ and kinetic energy loss Δ*E*_k_ as a function of the initial impact velocity *V*_0_ of the nanoprojectile collided with single layer graphene, ranging from 1000 to 7000 m/s. The minus symbol (-) of the residual velocity denotes the restitution process whereas the plus symbol (+) represents the penetration process.

**Table S1** *V*_bl_ obtained from Recht-Ipson model, Rosenberg-Dekel laminated model and MD simulations.

| Number of layer | Recht-Ipson Model | Rosenberg- Dekel  laminated model | MD simulation |
| --- | --- | --- | --- |
| 1 | 2367 | 855 | 3390 |
| 2 | 3222 | 1529 | 3890 |
| 3 | 3820 | 2061 | 4230 |
| 4 | 4278 | 2862 | 4470 |
| 5 | 5160 | 4804 | 5250 |
| 10 | 7085 | 6824 | 6910 |


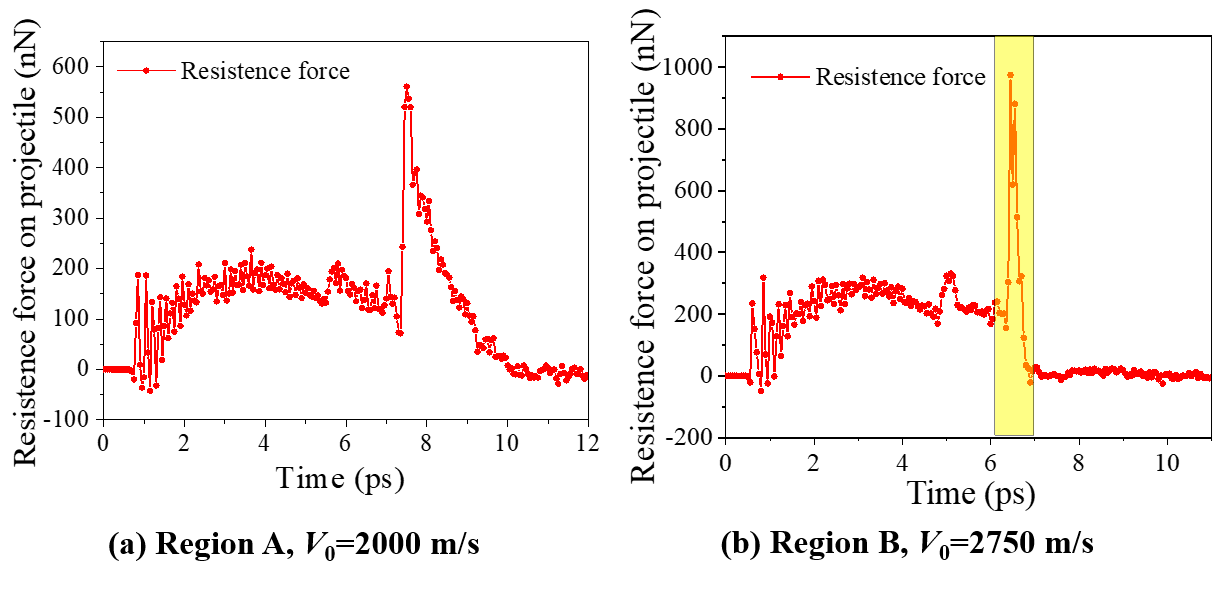
**(2)Energy conversion**

The detailed energy conversion will be analyzed from atomic level. We take the case of projectile of 4000 m/s impacting single-layer graphene as an example. The total energy consists of kinetic energy and potential energy. The variation of total energy of projectile (diamond) and target (graphene) as a function of time is shown in **Figure S2a**. As observed from Figure S2a, the energy dissipation of the projectile and the increase of graphene energy are almost exactly symmetrical, indicating that the energy loss of the projectile leads to the increase of graphene energy.

In the NVE ensemble, the energy conversion process should be met:

$\Delta E_{k,projectile}+\Delta E_{p,projectile}=\Delta E_{k,graphene}+\Delta E_{p,graphene}$ （2）

The energy conversion between the projectile and the projectile with respect to time is shown in Figure S2b.


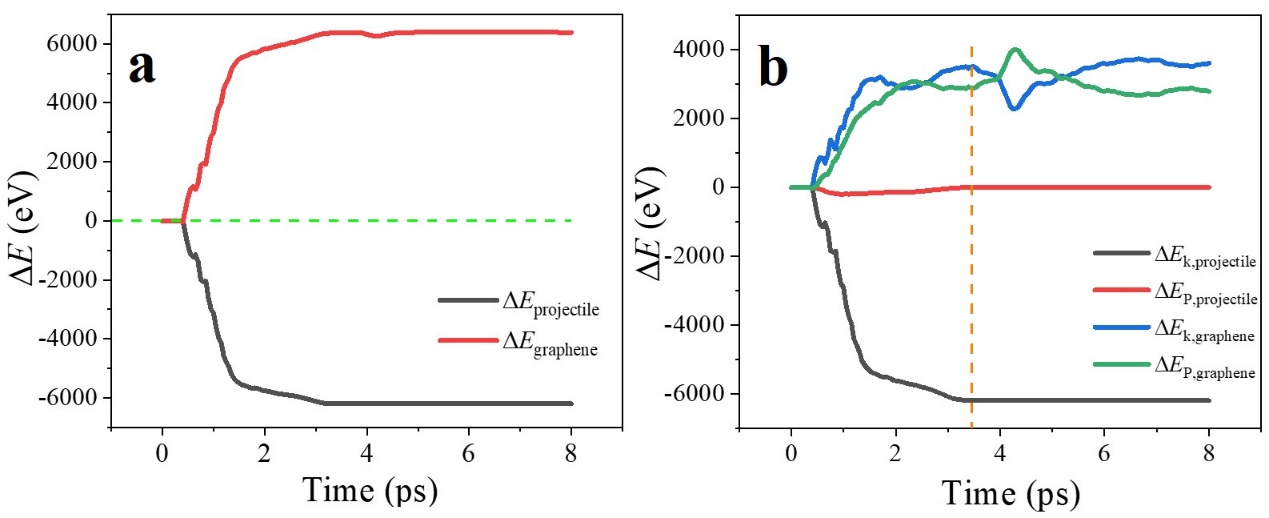


**Figure S2** Energy conversion between projectile and graphene during impact.

As observed from Figure S2b, with the increase of time, both the potential and kinetic energy of graphene increase while both the potential and kinetic energy of projectile decrease. The initial potential energy of projectile, close to zero, almost can be ignored. It can be seen that the loss of projectile kinetic energy mainly contributes to the reduction of projectile energy. Because it is a rigid projectile, the change in the potential energy of the projectile is mainly caused by the interaction between the C-C atoms, and there is almost no change in potential energy of projectile. After reaching 3.449 ps, it can be seen that the change between the potential energy and kinetic energy of graphene is almost symmetrical, indicating that the energy of graphene is only transforming between its internal potential energy and kinetic energy. Meanwhile, the kinetic energy and potential energy of the projectile do not change any more. Note that 3.449 ps is exactly the moment when the projectile leaves the graphene. After that, the projectile and graphene no longer interact with each other, and each acts as an independent system, only performing energy exchange inside.

In other words, part of the kinetic energy loss of projectile converts into the potential energy of graphene; another part of the kinetic energy loss of projectile convers into the kinetic energy of graphene, which can be reflected from the variation of the monitored temperature of graphene as shown in **Figure S3**. The variation of temperatures of both the projectile and graphene at 4000 m/s has been monitored and the results are shown in the following Figure S3.

It is known that the temperature is positively related to the kinetic energy:

$E_{k}=\frac{\dim}{2}N\text{k}T,$ （3）

where *E_k_* = total kinetic energy of the group of atoms (sum of 1/2 m v^2), dim = 2 or 3 = dimensionality of the simulation, *N* = number of atoms in the group, k = Boltzmann constant, and *T* = temperature.

**Figure S3** The variation of the temperatures of graphene and the projectile as a function of time.

It can be seen that at the beginning of the collision, the temperature of both the graphene and the projectile increase with increasing the simulation time. After the projectile is separated from the graphene, the temperature of the projectile tends to be stable whereas the temperature of the graphene is still fluctuating. Therefore, based on the above analysis, from the perspective of energy conversion, the reduction of the kinetic energy of the projectile is converted into the kinetic energy of graphene (the kinetic energy of the lost atoms, the oscillation of the atoms) and the increase of the potential energy (deformation energy, bond energy, angular energy, etc.). The temperature rise caused by the impact can also be observed.

1. **Interaction forces between projectile and target**

The interaction forces between the diamond nano-projectile and the single-layer graphene membrane at different initial impact velocities of 2000, 2750 and 3500 m/s, corresponding to the phenomena of “ricochet without damage”, “ricochet with damage” and “perforation”, respectively, have been displayed in **Fig. S4a**, **S4b** and **S4c**, respectively.

As shown in Fig. S4a, with the increase of the simulation time, the resistant force exerting upon the projectile first increases gradually and then decreases slightly, followed by a sudden hike to a peak of approaching 575 nN and subsequent drastic drop. Likewise, the resistance force as shown in Fig. S4b follows the almost identical trend to Fig. S4a, but the peak force attained of about 1000 nN is much larger. In contrast, the resistant force in Fig. S4c generally increases with the increase of simulation time until reaching a peak value of about 500 nN, followed by a sudden drop.

**Figure. S4** Resistance force exerting on projectile by the single layer graphene at the impact velocity of (a) 2000 m/s, (b) 2750 m/s, (c) 3500 m/s.

1. **Damage morphology**

Considering the hole’s tortuous boundary curve and polygon shape, the shape factor S_11_ is given by Tu et al,[^9^](#_ENREF_9) as follows:

(4)

where S_11_, *A*, *P* are the shape parameter, the area and circumferential length of hole, respectively. *A* and *P* of the hole left prior to the detachment were calculated using **ImageJ2x** image processing software.

The corresponding relationship between the value of S_11_ and the regular polygon is shown in the **Table S2**. For single-layer graphene membrane under different impact velocities, the morphology parameters P, A and S_11_ have been listed in **Table 1** and individually shown in **Fig. S5**.

The characteristic time is selected as the end of penetration when the projectile is detached from the membrane, we can measure the morphological quantities *A* and *P* of the damaged hole with the assistance of ImageJ2x image processing software. Then according to the quantitative formula listed above, the hole quantification S_11_ can be obtained. The corresponding relationship between the value of S_11_ and the regular polygon is shown in the **Table S2**.

**Table S2** Correspondence between the value of $S_{11}$and the shape.

| Hole shape | Circle | Regular hexagon | Regular pentagon | Regular  quadrangle | Regular  triangle |
| --- | --- | --- | --- | --- | --- |
| $S_{11}$ | 1.000 | 0.952 | 0.930 | 0.886 | 0.777 |

**Figure. S5**  Failure morphological parameters (*P*, *A* and *S_11_*) of the hole as a function of initial impact velocity ranging from 3500 to 5000 m/s for single layer graphene.

It’s apparent that as the impact velocity increases, the area *A* of the hole increases monotonically, in contrast, the boundary length *P* exhibits an upward trend after a decline, but the shape parameter always increases. The hole shape gradually transforms from triangle, quadrangle, pentagon and then to hexagon and the number of petals also gradually increases, all of these are consistent with the results reported [^10^](#_ENREF_10). In general, the morphology of the hole develops towards a polygon or even a circle. Likewise, the same quantitative analysis has also been applied multi-layer graphene but at slightly different impact velocities with an interval of 500 m/s. The corresponding calculated parameters have been listed in **Table S3** and the corresponding morphologies of the final hole are demonstrated in **Fig. S6**. As compared with single layer, the discrepancy lies in the fact that with the increase of the number of layers, the hole shape at the initial impact velocity (close to *V*_bl_) varies from triangle-like to quadrilateral and then tends to be polygonised. This probably can be explained from the steric hindrance. For the case of the single-layer graphene, the graphene membrane is prone to be damaged perpendicularly; in contrast, for the case of multi-layers, the ductile deformation is impeded by the bottom layer and the kinetic energy loss is in favor of transferring from perpendicular direction to the circumferential direction.

**Table S3** Morphological parameters *P*, *A* and *S*_11_ for single layer and multi-layer graphene membranes.

|  | *V*_0_ (m/s) | *A*/nm^2^ | *P*/nm | S_11_ |
| --- | --- | --- | --- | --- |
| Single-layer | 3500 | 17.286 | 19.590 | 0.752 |
|  | 4000 | 13.980 | 16.239 | 0.816 |
|  | 4500 | 24.412 | 19.645 | 0.891 |
|  | 5000 | 27.081 | 19.332 | 0.933 |
| Double-layer | 4000 | 14.953 | 17.391 | 0.788 |
|  | 4500 | 16.472 | 17.294 | 0.830 |
|  | 5000 | 25.465 | 19.052 | 0.938 |
|  | 5500 | 27.531 | 19.305 | 0.962 |
| Triple-layer | 4500 | 14.715 | 15.228 | 0.881 |
|  | 5000 | 17.603 | 16.712 | 0.889 |
|  | 5500 | 21.742 | 17.523 | 0.943 |
|  | 6000 | 26.071 | 18.803 | 0.962 |
| Quadruple-layer | 5000 | 16.051 | 16.217 | 0.875 |
|  | 5500 | 19.523 | 17.012 | 0.921 |
|  | 6000 | 21.058 | 17.574 | 0.942 |
|  | 6500 | 23.261 | 17.732 | 0.964 |

**
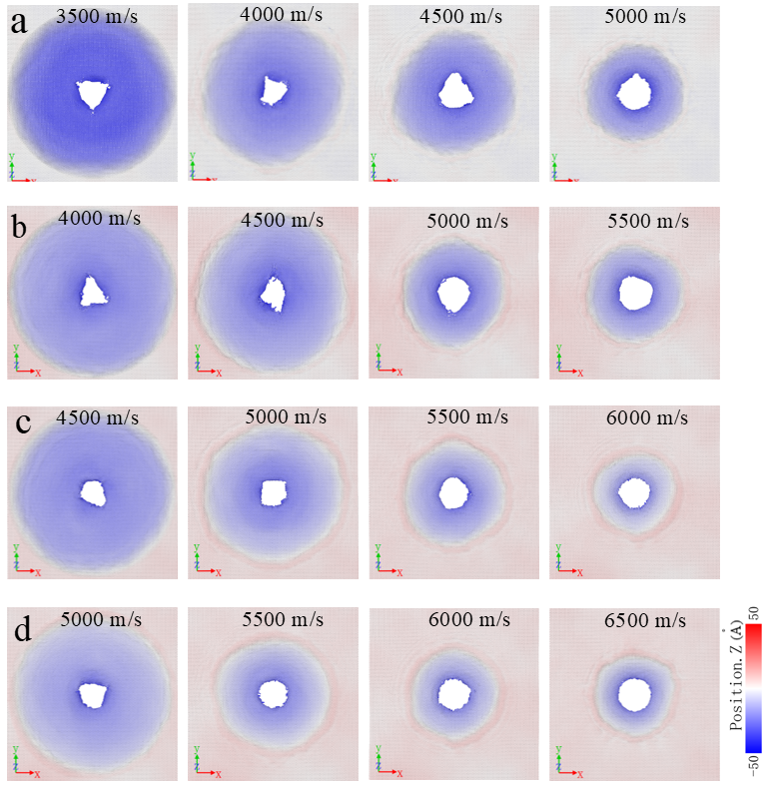
**

**Figure. S6** The top view of the holes with different number of layers at the characteristic moment with corresponding velocities. (a)Single-layer, (b) Double-layer, (c) Triple-layer, (d) Quadruple-layer. Positions are identified and visualized by Ovito Version 2.9.0 (https://www.ovito.org/).

1. **Depth of penetration**

In the theoretical models, such as F-W model[^11^](#_ENREF_11) and R-D model[^12^](#_ENREF_12), which are widely used in describing the penetration depth, the requirement for the shape of the projectile is hemispherical nosed projectile. Based on the principle of equal cross-sectional area and mass, the 6nm rigid spherical projectile is equivalent to hemispherical nosed projectile, as shown in **Figure S7**:


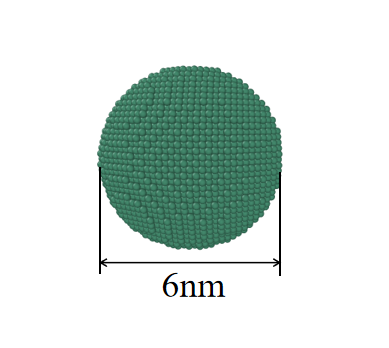

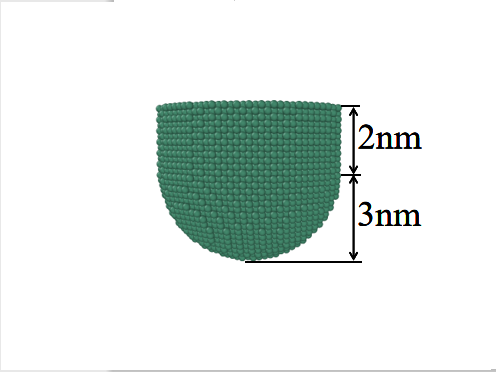


**Figure. S7** Spherical projectile and its equivalent hemispherical projectile.

1. ***Z_max_ impact displacement and total time of impact***

The definition of ‘contact’ is identical to the previous works and corresponds to the point where the first non-zero value of force exerting on the projectile by the graphene appears. The total impact displacement is the distance of the projectile travelling from the initial contact point in the approach process to the final contact point at the instant of initiated restitution where the kinetic energy becomes zero or perforation along the Z-axis. Correspondingly, the total time of impact is the time interval from the instant of the initial contact to that of the final contact between the projectile and graphene. Fig. S1 shows the resistant force exerting on the projectile by the single layer graphene at 4200 m/s, which first initiates from almost zero value and then fluctuates seriously, followed by decreasing gradually until reaching zero again. In this case, the total time of impact is about 2.8 ps.

As observed from **Fig. S8a**, it is easily understood that the impact displacement in the phase of restitution without damage to the graphene increases with increasing the initial impact velocity (the higher the impact velocity, the greater the deformation of graphene) and then increases to a peak value corresponding to the phase of restitution with the failure of graphene, followed by a gradual decrease in the phase of penetration. Note that the higher number of layers leads to the smaller impact displacement in the phase of restitution whereas the opposite trend is observed from the phase of penetration. When it comes to the total time of impact (**Fig. S8b**), it first decreases to a local minimum in the phase of restitution, followed by experiencing a ‘pop-in’ (i.e., increasing to a local peak) and subsequent sudden drop in the phase of penetration. Note that more layers lead to longer total time of impact and broader transition region, but this phenomenon becomes weakened in the phase of penetration especially at high impact velocity.


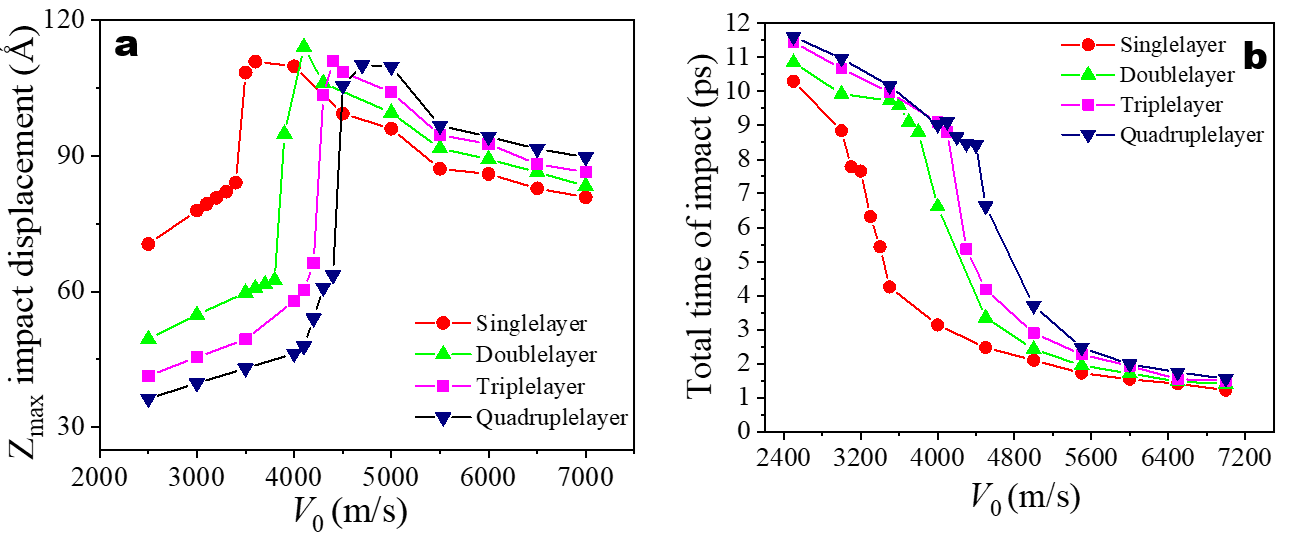
**Figure. S8** Total impact displacement (a) and total time of impact (b) as a function of initial impact velocity of the projectile collided with single/multi-layer graphene, ranging from 2500 to 7000 m/s.

1. **Surface effect**

The results we reported are directly obtained from a single MD simulation, instead of taking the average value after repeated simulations. Of course, we have repeatedly simulated a certain case and found that the results are basically the same. Of course, there are other factors that can affect the simulation results, such as the surface roughness of the nanospheres. This factor was also explored in our previous work on the high-speed impact of two nanospheres. We have further explored the influence of nanoprojectile's surface roughness on the simulation results. We rotate the diamond nanospheres along the Z axis by 45°, 90°, 135°, and 180°. Figure S9 shows the morphology of the nanospheres after rotating at different angles. We use nanospheres rotated at different angles as projectiles to simulate the impact of graphene to explore the impact of the difference in surface roughness. We selected two different impact velocities for simulation (2000 m/s and 5000 m/s), and the results are shown in Figure R6. It can be found that at the two impact velocities, the fluctuations in the value of the residual velocity of the projectiles with different rotation angles after impact are all within 1%. This indicates that the surface roughness of nanospheres has a small effect on impact simulations, which is similar to the conclusions of previous work[^13^](#_ENREF_13), [^14^](#_ENREF_14).


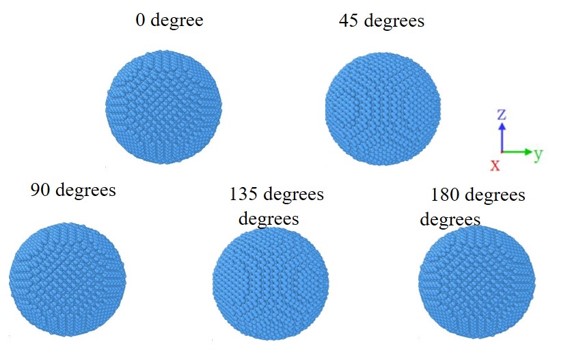


**Figure. S9** The morphology of the projectile after rotating different angles along the Y axis. (a) 0°, (b) 45°, (c) 90°, (d) 135°, (e) 180°.

**a**

**b**

**Figure. S10** The relationship between the residual velocity and the rotation angle of the projectile.

The present MD simulation is “experimental”. It is a type of numerical experiments which are equivalent to physical experiments if achievable. Experimentation would suffer from errors. Although reproducible for single one simulation, numerical experiments also have errors if considering the surface effect.

1. **Effect of rigid assumption**

In order to further explore the effect of the “rigid” assumption, as suggested by the reviewer, the same potential of AIREBO potential have been used herein to simulate the projectile impact with one single-layer graphene by monitoring the residual velocity, as shown in **Figure S11**. (It should be noted that AIREBO potential itself contains LJ, but in the previous manuscript, new parameters for LJ potenial are used and the LJ fucntion in AIREBO was turned off.)

It is found that the variation trend of residual velocity of projectile and ballistic limiting velocity of monolayer graphene under the two schemes are basically consistent with each other, But because of the “rigid” and “non-rigid” assumption, the residual velocities are different. However, this has little influence on the evaluation of the ballistic limiting velocity.

**Figure 11** Residual velocity *V_r_* as a function of the initial impact velocity *V*_0_ of the nanoprojectile collided with single layer graphene under two kinds of potentials.

In addition, the indexes of penetration depth formed by impacting 10-layer graphene under two potential functions are further observed, and they are compared with the A-T model in penetration eroding model[^15^](#_ENREF_15), A-T model：

$$\frac{P}{L}\text{=}\frac{\rho_{\text{p}}}{\rho_{t}}\ln(1+\frac{\rho_{t}V^{2}}{2R_{t}}),$$

(5)

where Rt is the target resistence to penetration, ρp and ρt are the density of projectile and target (ρ_p_=3.5 g/cm3, ρ_t_=2.2 g/cm3), L is the effective length of projectile.

For the spherical projectile, Tate model coefficient can be obtained as follows:

$\text{R}_{t}\text{=}\sigma_{Yt}[\frac{2}{3}+\ln(\frac{0.57E_{\text{t}}}{\sigma_{Yt}})],$ (6)

where E_t_ is the the Young's modulus of target, σ_Yt_ is the flow stress of target. Add parameter data，Et =1TPa，σ_Yt_ =140Gpa [^16^](#_ENREF_16), then Rt =290 Gpa. We get:

Under the two schemes, the penetration depth resulted from projectile impact with 10-layer graphene is shown in **Figure S12**:

**Figure S12** The penetration depth formed by impacting 10-layer graphene under two potential schemes.

It is found that under the condition of non-rigid, with the increase of the impact velocity, the penetration depth gradually tends to match the prediction of A-T model. Certain atomic loss may occur and large deformation has been created at high impact speed, similar to “eroding” in the penetration.

Significant difference can be clearly observed in terms of penetration depth. This is readily understood since these two assumptions correspond to rigid penetration and deforming penetration. The corresponding continuum theories are also different. Our paper mainly is associated with the rigid penetration, with a focus on the impact of rigid projectile with the graphene. But the reviewer’s concerns provide further exploration and improvement direction for our follow-up work.

1. **Effect of “free” or “ fixed” boundary conditions**

In order to further explore the influence of boundary conditions，we make more efforts to build two models of 50 layers of graphene: (a) First plan, the entire graphene membrane is subjected to periodic boundary conditions in the X, Y, Z three dimensional directions and all the outmost atoms of the 4 planes along the Z direction are “free”; (b) Second plan, the entire graphene membrane is subjected to periodic boundary conditions in the X, Y, Z three dimensional directions but outmost atoms of the 4 planes along Z direction are “fixed”. This setting of fixed boundary condition is analogous to the one in our manuscript. The combination of 50 layers of graphene with the projectile impact model in **Figure S13**:


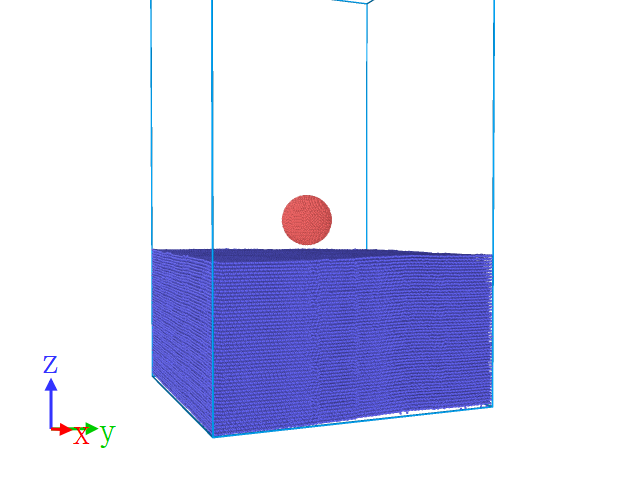


**Figure S13** Diagram of 50 layers of graphene combined with projectile impact model.

Due to the limitation of computation cost and efficiency, the impact velocity of 5000 m/s is selected as an example to compare the remaining velocity and the force exerting on the projectile in the whole impact process as a function of time under the two plans, as shown in the **Figure S14**. It can be found from the **Figure S14** that the setting of the boundary around the two X, Y directions has little influence on the change of the residual velocity of the projectile and force exerting on the projectile in the whole impact process.

**Figure S14** The variation of residual velocity and force exerting on the projectile as a function of time under the free or fixed boundary conditions.

Then we turn our attention to the comparison of depth of penetration of the projectile under the two boundary conditions. The maximum Z-displacement of the projectile impacting 50 layers of graphene at 5000 m/s is obtained, which is 27.2(Å) and 26.9(Å), respectively, under the free and fixed conditions. The snapshots of the maximum Z-displacement at the identical time is shown as follows in **Figure S15**:


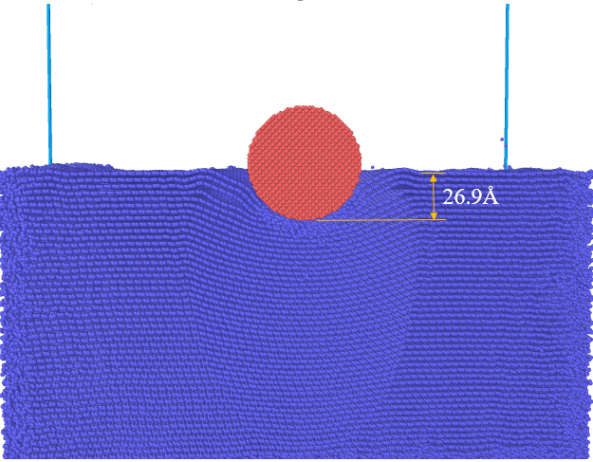

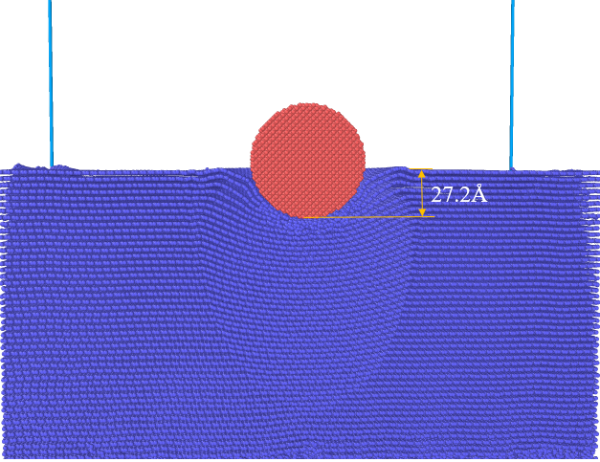


**Figure S15** The screenshot of the maximum Z-displacement time. (a) First plan, (b) second plan.

In summary, the difference between the two indices under the above two boundary schemes is within 5%, which indicates that the setting of fixed boundary around the model and the setting of periodic boundary around the model have little influence on impact results. The setting of “free” or “fixed” boundary conditions will affect the *shockwave reflecting* , but since this work focus on the penetration behaviors of multilayer graphene and such setting has little influence on the points we are interesting in this work. In addition, in many simulations of projectiles impacting graphene, fixed boundary conditions of graphene have been adopted, to explore the dynamic penetration behaviors of graphene using MD simulations [^3-8^](#_ENREF_3).

**References**

1. Lee, J. H., Loya, P. E., Lou, J., Thomas, E. L., Dynamic mechanical behavior of multilayer graphene via supersonic projectile penetration. *Science*. **346**, 1092-1096(2014).

2. Meng, Z. X., Singh, A., Qin, X., Keten, S., Reduced ballistic limit velocity of graphene membranes due to cone wave reflection. *Extreme Mech. Lett.* **15**, 70-77(2017).

3. Xia, K., Zhan, H. F., Hu, D. A., Gu, Y. T., Failure mechanism of monolayer graphene under hypervelocity impact of spherical projectile. *Sci Rep*. **6**, 10(2016).

4. Dong, Y. N., He, Y. Z., Wang, Y., Li, H., A theoretical study of ripple propagation in defective graphene. *Carbon*. **68**, 742-747(2014).

5. Inui, N., Mochiji, K., Moritani, K., Actuation of a suspended nano-graphene sheet by impact with an argon cluster. *Nanotechnology*. **19**, 7(2008).

6. Kumar, N., Poonia, A. K., Kumar, A., Thapa, K. B., Pandey, G. N., Suthar, B., Analysis of the Impact of Graphene Coating on Reflectivity of a Silicon Substrate for Optoelectronic Devices, 3rd International Conference on Condensed Matter & Applied Physics (ICC), Amer Inst Physics, Bikaner, INDIA, 2019.

7. Hosseini-Hashemi, S., Sepahi-Boroujeni, A., Sepahi-Boroujeni, S., Analytical and molecular dynamics studies on the impact loading of single-layered graphene sheet by fullerene. *Appl. Surf. Sci.* **437**, 366-374(2018).

8. Yoon, K., Ostadhossein, A., van Duin, A. C. T., Atomistic-scale simulations of the chemomechanical behavior of graphene under nanoprojectile impact. *Carbon*. **99**, 58-64(2016).

9. Tu, X., B, Wang, S, J., Particle shape descriptor in digital image analysis. *Chinese Journal of Geotechnical Engineering*. **26**, 259-262(2004).

10. Haque, B. Z., Chowdhury, S. C., Gillespie, J. W., Molecular simulations of stress wave propagation and perforation of graphene sheets under transverse impact. *Carbon*. **102**, 126-140(2016).

11. Forrestal, M. J., Warren, T. L., Penetration equations for ogive-nose rods into aluminum targets. *Int. J. Impact Eng.* **35**, 727-730(2008).

12. Xiao, Y. K., Wu, H., Fang, Q., Zhang, W., Kong, X. Z., Hemispherical nosed steel projectile high-speed penetration into aluminum target. *Mater. Des.* **133**, 237-254(2017).

13. Sun, W. F., Zeng, Q. H., Yu, A. B., Kendall, K., Calculation of Normal Contact Forces between Silica Nanospheres. *Langmuir*. **29**, 7825-7837(2013).

14. Jiang, J., Chen, P. W., Sun, W. F., Monitoring micro-structural evolution during aluminum sintering and understanding the sintering mechanism of aluminum nanoparticles: A molecular dynamics study. *J. Mater. Sci. Technol.* **57**, 92-100(2020).

15. Anderson, C. E., Analytical models for penetration mechanics: A review (vol 108, pg 3, 2017). *Int. J. Impact Eng.* **115**, 120-120(2018).

16. Lee, C., Wei, X. D., Kysar, J. W., Hone, J., Measurement of the elastic properties and intrinsic strength of monolayer graphene. *Science*. **321**, 385-388(2008).
